# Safety and Efficacy of Cariprazine in a Subset of Patients with Schizophrenia: A Phase IV Study

**DOI:** 10.1192/j.eurpsy.2026.12141

**Published:** 2026-07-31

**Authors:** C. S. Jilowa, N. Vemana, M. Bhirud, A. Dharmadhikari, D. Chaudhari, Y. Patel, P. Dasud, R. Khandelwal, S. K. Padhy, S. Gopal, L. Lakhwani, S. saha, D. Patil, S. Behera, S. Pandit, P. Ghadge, S. Mehta

**Affiliations:** 1 Jawahar Lal Nehru Medical College, Ajmer; 2 Excel Hospital, Secunderabad; 3 Dhadiwal Hospital Incoalition with Shreeji Healthcare, Nashik; 4 Shree Aashirwaad Hospital, Dombivili; 5 GSVM Medical College, Kanpur; 6 VS General Hospital, Ahmedabad; 7 Calida Psychiatric Hospital, Raigad; 8 Apex hospital Pvt Ltd, jaipur; 9 All India Institute of Medical Sciences, Bhubaneswar; 10 S.P. Medical College & A.G. of Hospitals, Bikaner; 11 Sun Pharma laboratories limited, Mumbai, India

## Abstract

**Introduction:**

Cariprazine is an atypical antipsychotic drug, demonstrating efficacy for the treatment of schizophrenia. This pharmacologic profile allows cariprazine to effectively treat the broad range of schizophrenia symptoms including positive symptoms (like delusions and hallucinations), negative symptoms (such as emotional withdrawal and social apathy), and cognitive impairments. A Phase IV, prospective, multi centre, single arm study was conducted to evaluate safety and efficacy of cariprazine in Indian patients with Schizophrenia.

**Objectives:**

The Safety and Efficacy of Cariprazine capsules was evaluated in the subset of patients with with schizophrenia.

**Methods:**

Patients diagnosed with schizophrenia with or without psychotic symptoms at least 1 year prior, based on DSM-V criteria, were enrolled in the study with the Positive and Negative Syndrome Scale (PANSS) 80-120 (both inclusive). A total 97 patients, in the age range of 18 to 65 years, received Cariprazine starting dose 1.5 mg with further up titration to 3 to 6 mg for 6-weeks Primary Endpoint was safety evaluation and Secondary Endpoints were change PANSS score (total, positive subscale and negative subscale) Clinical global impression-Severity (CGI-S) score. **[Clinical Trial Registration: [CTRI/2023/05/052793]**

**Results:**

A total of 39 TEAEs were reported in 29, patients, Insomnia was the most common TEAE 12 events, followed by constipation, headache, sleep disorder, and night sweats (3 events each). All events were grade 1 (mild) and resolved.

With cariprazine treatment continued, there is gradual improvement in study patients based on PANSS total scores change (Mean±SD) constantly from Day 7 (-3.70 ±4.99) to Day 42 (-32.94 ±16.25). Similarly Positive and negative subscale scores (Mean±SD) were changed by Day 42 -9.79 ± 6.19 and -6.02 ± 4.30, respectively. CGI-S scores (Mean±SD) showed consistent improvement, decreasing from -0.20±0.40 at Day 7 to -1.55±1.02 at Day 42.

**Conclusions:**

Cariprazine capsules demonstrated efficacy in managing the clinical symptoms of schizophrenia This drug may serve as a therapeutic option, potentially reducing symptom severity and enhancing overall mental well-being. The study treatment was found to be safe and well tolerated.

**Disclosure of Interest:**

None Declared

